# Influences of Combined Treatment by Cement Slurry and Methyl Sodium Silicate Solution on Recycled Coarse Aggregate and Recycled Aggregate Concrete

**DOI:** 10.3390/ma18163832

**Published:** 2025-08-15

**Authors:** Jinming Yin, Aihong Kang, Changjiang Kou

**Affiliations:** 1 Taizhou Institute of Science and Technology, Nanjing University of Science and Technology, Taizhou 225300, China; 2 College of Civil Engineering and Transportation, Yangzhou University, Yangzhou 225100, China; kahyzu@163.com (A.K.); changjiang.kou@yzu.edu.cn (C.K.)

**Keywords:** recycled coarse aggregate (RCA), recycled aggregate concrete (RAC), combined treatment method, methyl sodium silicate, cement slurry, spraying method

## Abstract

The poor quality of recycled coarse aggregate (RCA), particularly its high water absorption and low strength, has long restricted the development of recycled aggregate concrete (RAC). In this study, a novel combined spraying treatment method integrating cement slurry and a methyl sodium silicate (MSS) solution was proposed to improve the comprehensive performance of RCA. The effects of the treatment on RCA properties, including crushing value, water absorption, dynamic water absorption, apparent density, micromorphology, and contact angle, were systematically investigated. Furthermore, the treated RCA was incorporated into concrete to evaluate the mechanical strength, water absorption, and interfacial transition zone (ITZ) properties of the resulting RAC. The results indicated that cement slurry treatment alone significantly reduced the crushing value of the RCA by 30.1% but had little effect on water absorption. Conversely, MSS solution treatment reduced RCA water absorption by 29.6% without affecting its strength. The combined spraying method successfully enhanced both strength and water absorption performance. When applied in the RAC, cement slurry-treated RCA improved compressive and splitting tensile strengths, while MSS-treated RCA notably reduced water absorption. RAC prepared with combined-treated RCA achieved further strength improvement, and although its water absorption was not as low as that of MSS-only treated RAC, it still showed a substantial decrease compared to untreated RCA. Nanoindentation and microstructural analyses revealed that MSS enhanced the ITZ by forming a hydrophobic molecular film and reacting with new mortar, inhibiting water transport and improving RAC durability. An optimal MSS concentration of 10% was identified for achieving the best combined performance in strength and durability.

## 1. Introduction

China generates over 3 billion tons of construction and demolition waste (CDW) annually, creating significant challenges in terms of land occupation and environmental pollution [1,2,3]. Concurrently, the rapid development of the construction industry has led to the intensive exploitation of natural resources, particularly aggregates and sand, further exacerbating ecological concerns [4]. The recycling of CDW into recycled aggregate (RA) presents a viable solution to simultaneously address these pressing environmental and resource challenges [5,6,7,8].

However, the presence of adhered mortar significantly compromises the quality of recycled aggregate (RA), resulting in inferior properties compared to natural aggregate (NA), including higher crushing values, increased water absorption, and reduced density [9,10,11,12,13,14]. These deficiencies consequently lead to poorer performance in recycled aggregate concrete (RAC), manifested through reduced workability, diminished mechanical strength, and compromised durability [15,16].

To improve the performance of RA and RAC, numerous studies have been carried out. Current research primarily focuses on two approaches: removing old mortar and strengthening existing mortar. In addition, research on the application of machine learning and artificial intelligence has gradually emerged, primarily focusing on optimizing RCA reinforcement strategies, designing RAC mix proportions, and predicting the mechanical and durability performance of recycled concrete [17,18,19,20,21,22,23]. As recycled aggregate (RA) serves as the primary component of recycled aggregate concrete (RAC), enhancing RA performance has become a crucial measure for expanding its applications, consequently attracting increasing research attention. To remove the attached old mortar, acid dissolution [24,25] and mechanical abrasion [26,27] are the primary methods employed. To strengthen the old mortar, cement-based slurries [28,29], sodium silicate [14,30], nanomaterials [31,32], CO_2_ [33,34], and bacteria [35,36] are usually employed. While these methods effectively enhance RA properties, they introduce several limitations that must be considered. Environmental risk should be taken into account when using an acid solution. Additional energy is required, which leads to the emission of CO_2_ when using the abrasion method. CO_2_ and bacteria treatment methods usually require a long time. Therefore, mortar enhancement through solution-based treatment emerges as a promising alternative approach.

Numerous studies have explored slurry-based treatments for enhancing recycled aggregate (RA) properties. Olofinnade et al. [37] demonstrated that coating recycled coarse aggregate (RCA) with a metakaolin-cement slurry significantly improved concrete compressive strength and splitting tensile strength, while reducing water absorption. Alternative biological approaches have shown promise, as evidenced by Zhang et al. [38], where bacterial treatment producing biogenic CaCO_3_ reduced RA water absorption.

Composite cementitious systems have yielded particularly notable results. Xu et al. [39] developed a ternary slurry incorporating cement, silica fume, and fly ash, which when applied via pre-coating enhanced the RAC strength properties. Yin et al. [40,41] systematically compared cement slurry and water glass treatments, finding that while cement spraying reduced the RCA crushing value (though with minimal impact on water absorption), water glass application improved both crushing resistance and water absorption.

Advanced modification techniques have achieved even more substantial improvements. Li et al. [42] developed a sodium silicate-silane compound solution that decreased RA water absorption while enhancing RAC performance. Nanotechnology applications have shown particular promise, with Zhao [43] reporting 33.3% and 30.8% reductions in water absorption and crushing value, respectively, through nano-SiO_2_ slurry treatment, accompanied by significant elastic modulus improvements in RAC. Similarly, Kou et al. [44] achieved 20.49% and 30.02% reductions in crushing value and water absorption using tetraethoxysilane solution treatment.

While cement-based slurries remain a widely used strengthening solution due to their cost-effectiveness and ready availability, they demonstrate limited effectiveness in reducing the water absorption of both recycled aggregate (RA) and recycled aggregate concrete (RAC). The development of composite treatment methods for RCA remains an ongoing challenge, as the effects and underlying mechanisms on both RCA and RAC performance vary significantly depending on the specific treatment protocols employed, warranting further in-depth investigation. To address this limitation, we developed an innovative combined treatment approach employing cement slurry and a methyl sodium silicate (MSS) solution, applied through an efficient spraying technique that optimizes material usage and processing time [40,41]. This study comprehensively evaluated the performance enhancements in both RA and RAC, with particular emphasis on characterizing the interfacial transition zone between the treated RA and new mortar to elucidate the underlying strengthening mechanisms of the combined treatment.

## 2. Materials and Methodology

### 2.1. Materials

#### 2.1.1. RCA

In this study, recycled coarse aggregate (RCA) was supplied by Yangzhou Huimin Renewable Resources Co., Ltd., Yangzhou, China. The particle size of the RCA ranged from 9.5 mm to 19 mm, with an apparent density of 2687.3 kg/m^3^, a crushing value of 15.27%, and a 24 h water absorption of 5.81%. According to “Recycled Coarse Aggregate for Concrete” (GB/T 25177-2010) [45], the employed RCA was classified as comprehensive Grade III, primarily due to its water absorption.

#### 2.1.2. Cement

An ordinary Portland cement (P.O 42.5), manufactured by Taizhou Yangwan Hailuo Cement Co., Ltd. (Taizhou, China), was used in this study, which is widely applied in the construction industry, readily available, and cost-effective. The chemical compositions are listed in Table 1.

#### 2.1.3. Methyl Sodium Silicate

**Figure 1:**
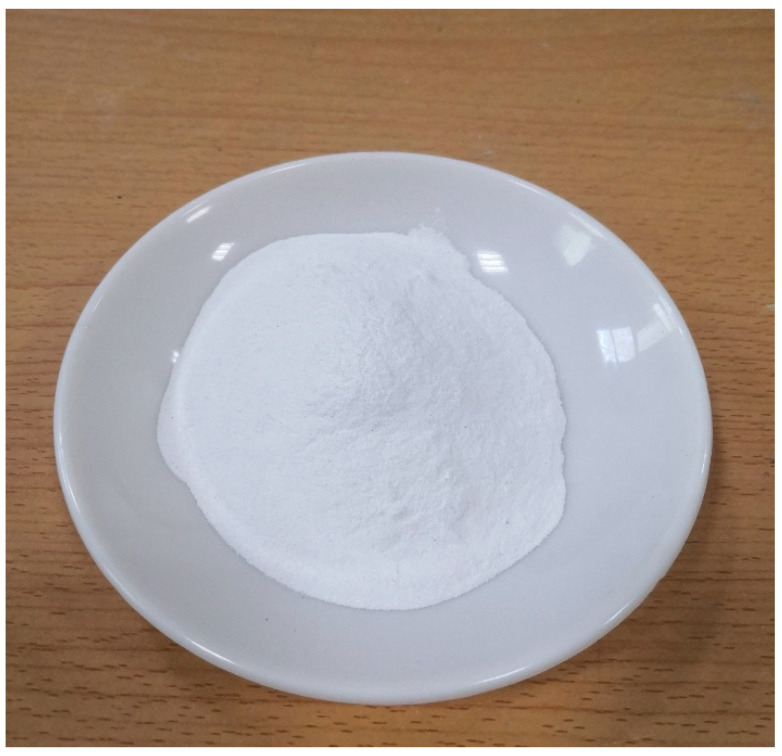
Methyl sodium silicate powder.

Methyl sodium silicate (MSS) is a waterproof material which can penetrate into concrete and generate a water-proof layer on the surface of the concrete and capillary pore [46]. MSS powder supplied by Jinan Xingchi Chemical Co., Ltd. (Jinan, China) was used, as shown in Figure 1.

### 2.2. Test Methods

#### 2.2.1. Treatment Method of RCA

According to our previous studies [40,41], the spraying method was employed to treat the RCA. First, the RCA was treated with cement slurry, and the ratio of water to cement (*w*/*c*) was 0.7, which has been shown to enhance the strength of RCA [40]. Second, the treated RCA was cured under moist conditions for 7 days, followed by air drying. Third, the air-dried RCA was treated with the MSS solution and then air dried. The combined treatment scheme is shown in Table 2.

**Figure 2:**
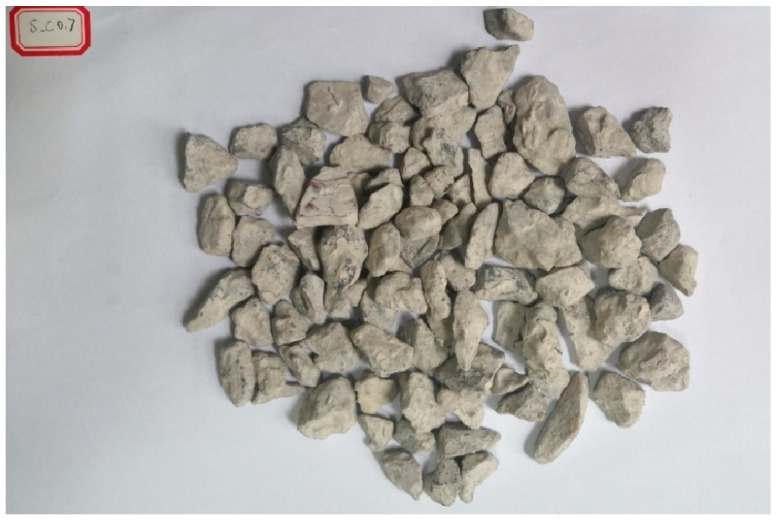
RCA treated by cement slurry.

**Figure 3:**
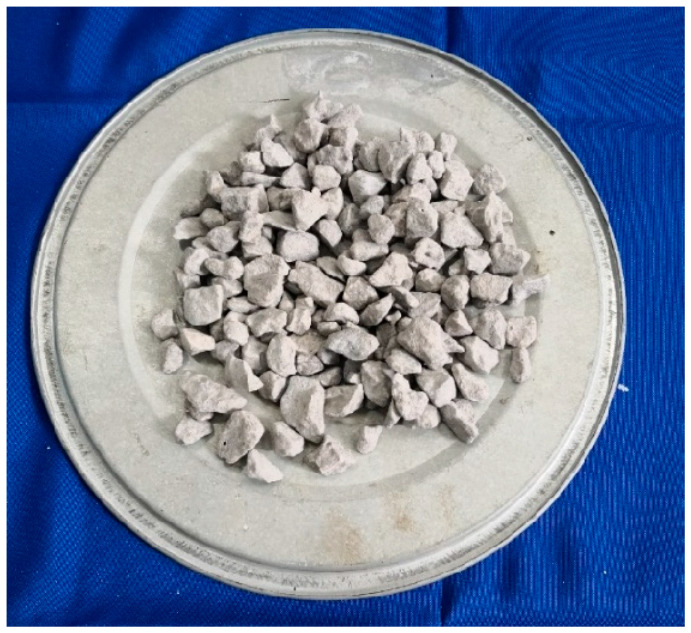
RCA treated with cement slurry and MSS solution.

After treatment with cement slurry, a layer of hardened cement paste formed on the RCA surface, as shown in Figure 2. The old mortar of the RCA was barely visible, and the pores and cracks were also filled, making the surface smoother. After treatment with MSS solution, no significant variations were observed between the treated and untreated RCA specimens., which meant that the MMS penetrated into the RCA, as shown in Figure 3.

#### 2.2.2. Performance Tests of RCA

(1) Apparent density, crushing value and water absorption

In this study, the apparent density, crushing value, and water absorption of RCA were tested according to [45] and “Pebble and Crushed stone for construction” (GB/T 14685-2011) [47].

(2) Dynamic water absorption

The evolution of water absorption during 24 h was tested according to [48,49]. At time *t*, the water absorption (*w*_at_) was determined using the following equations:

(1) $$w_{\mathrm{at}}\;=\;\frac{m_{t}-m_{s}}{m_{1}}\;\times \;100\%$$

(2) $$m_{s}=m_{24}-w_{a}\;\times \;m_{1}$$

where *m*_t_ is the weight of the RCA immersed in water at time *t*, g; *m*_1_ is the weight of the oven-dried RCA, g; *m*_24_ is the weight of the RCA immersed in water for 24 h; and *w*_a_ is the 24 h water absorption of the RCA.

(3) Water contact angle of old mortar

**Figure 4:**
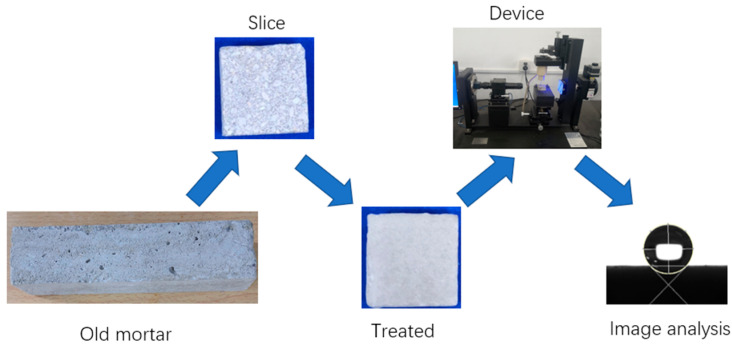
The test procedure of the contact angle.

The water contact angle was measured to assess the influences of different treatment methods on the hydrophilicity of the old mortar. First, mortar samples were prepared and stored at room temperature for more than 270 days. Second, the old mortar samples were cut into several slices. These slices were subsequently treated with cement slurry and MSS solution. Finally, the water contact angle was tested. The contact angle measuring device was produced by Shanghai Zhongchen Digital Technology Apparatus Co., Ltd. (Shanghai, China) and ImageJ (version, 1.53t) was used to analyze the contact angle. The test procedure is shown in Figure 4.

(4) Micromorphology and element analysis

The scanning electron microscope (SEM) was used to observe the micromorphology of the RCA, and the energy dispersive spectrometer (EDS) was also used to characterize the influence of the treatment method on the RCA. The GeminiSEM 300 was utilized in this study.

(5) Fourier transform infrared spectroscopy analysis

**Figure 5:**
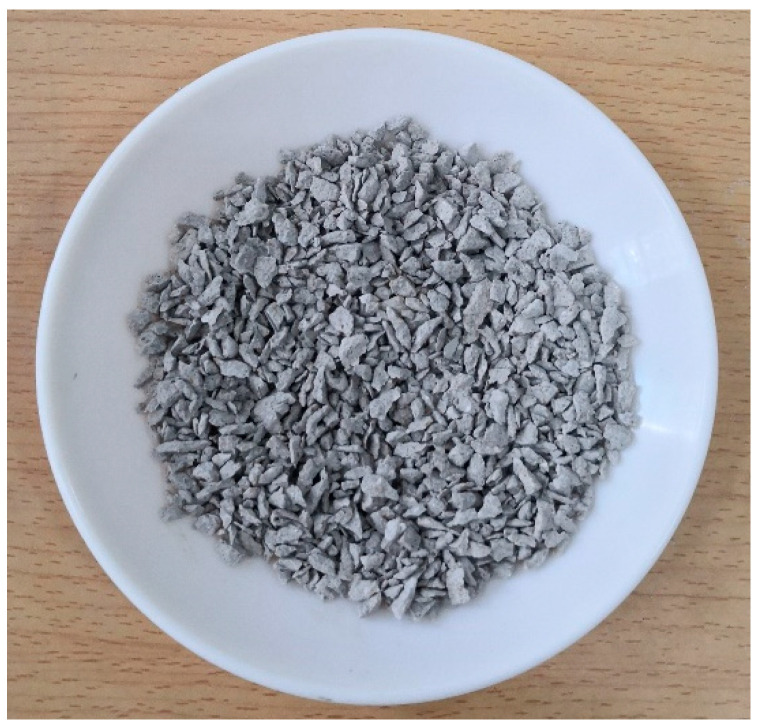
Crushed old mortar.

The Fourier transform infrared spectroscopy test (FTIR) was conducted to investigate the effects of the combined treatment method on the functional groups of the old mortar. First, the old mortar samples were crushed and the crushed old mortar (COM) was collected and sieved, as shown in Figure 5. The COM was then treated with cement slurry and the MSS solution. Finally, the treated COM was air-dried and ground into powder. The scanning range was 400–4000 cm^−1^ and the instrument was manufactured by PerkinElmer Inc. (Waltham, MA, USA)

#### 2.2.3. Preparation of Concrete

The performance of concrete prepared with different types of aggregates was also evaluated, and the mix proportions are listed in Table 3. The continuously graded aggregates were used. The crushing value, water absorption, and particle size of the natural coarse aggregate (NCA) were 8.15%, 0.46%, and 9.5–19 mm, respectively. The fine aggregate used was river sand, classified as medium sand. The natural aggregate (NA) was used directly, while the untreated RCA and cement slurry-treated RCA were used in a saturated surface-dry (SSD) condition. For the MSS solution-treated RCA and the combined-treated RCA, water was sprayed after MSS treatment to maintain the SSD condition before use. When preparing recycled aggregate concrete (RAC), NCA was completely replaced with RCA. The experimental design for the concrete mixtures is presented in Table 4.

#### 2.2.4. Performance Test of Concrete

(1) Mechanical strength

The compressive strength and splitting tensile strength were tested based on the “Standard for test methods of concrete physical and mechanical properties” (GB/T 50081-2019) [50]. The concrete sample size was 10 cm × 10 cm × 10 cm and the loading rates were 0.5 MPa/s and 0.05 MPa/s, respectively. The compressive strength and splitting tensile strength were determined by the following equations:

(3) $$\sigma_{c}\;=\;\frac{N_{max}}{100\;\times \;100}$$

(4) $$\sigma_{sp}=0.637\;\times \;\frac{F_{max}}{100\;\times \;100}$$

where $\sigma_{c}$ is the compressive strength, MPa; $N_{max}$ is the maximum load during the compressive strength test, N; $\sigma_{sp}$ is the splitting tensile strength, MPa; and $F_{max}$ is the maximum load during the splitting tensile strength test, N.

(2) Water absorption

The durability of concrete is related to its water absorption characteristics [7]. In this study, the water absorption of concrete was tested based on ASTM C1585-13 [51]. Cylindrical samples were used whose diameter and height were 100 mm and 50 mm, respectively. The absorption was determined using the following equation:

(5) $$I\;=\;\frac{m_{wt}}{a\cdot d}$$

where *I* is the absorption, mm; $m_{wt}$ is the mass increment of the sample at time *t*, g; *a* is the area of the sample contacting with water, mm^2^; and *d* is the density of water, g/mm^3^.

Straight lines were used to fit the relationship between *I* (mm) and the square root of *t* (s^0.5^). The slopes of the fitting lines within the first 6 h and after the first day were named as the initial rate of absorption (*S*_i_) and secondary rate of absorption (*S*_s_), respectively. The fitting equation is as follows:

(6) $$I\;=\;S\sqrt{t}\;+\;b$$

where *S* is the rate of absorption, mm/s^0.5^; *b* is the fitting parameter, mm.

(3) Nanoindentation

**Figure 6:**
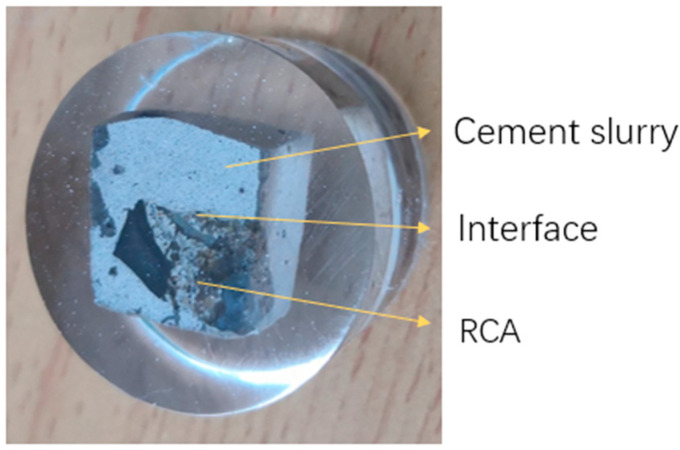
Nanoindentation sample.

Nanoindentation was employed to investigate the effect of different RCAs on the interfacial transition zone between the old mortar and the new mortar. First, the RCA was treated according to the procedures listed in Table 2. Then, a 0.5 *w*/*c* ratio cement slurry was prepared. The treated RCA was added to the slurry, and the specimens were cured for 28 days. After curing, the samples were cut, embedded in resin, and subsequently polished, as shown in Figure 6. The nanoindentation testing scheme is summarized in Table 5.

**Figure 7:**
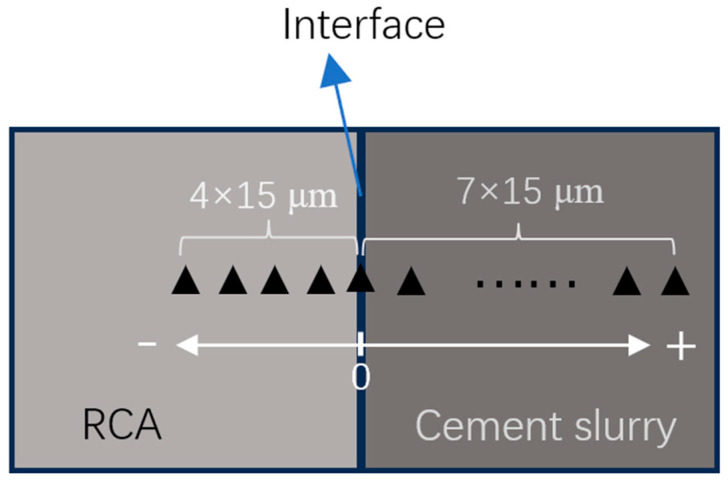
Arrangement of nanoindentation points.

A Nano Indenter G200, manufactured by Agilent Technologies, Inc. (Santa Clara, CA, USA), equipped with a Berkovich indenter, was used for the tests. Nanoindentation was performed at a controlled loading rate of 120 μN/s, with a maximum load of 1200 μN. The indentation points were arranged along the vertical direction across the interface, as illustrated in Figure 7.

**Figure 8:**
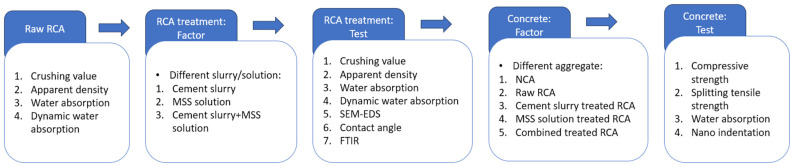
Technical roadmap.

The technical roadmap of this study is illustrated in Figure 8.

## 3. Results and Discussion

### 3.1. Results of RCA Tests

(1) Apparent density

**Figure 9:**
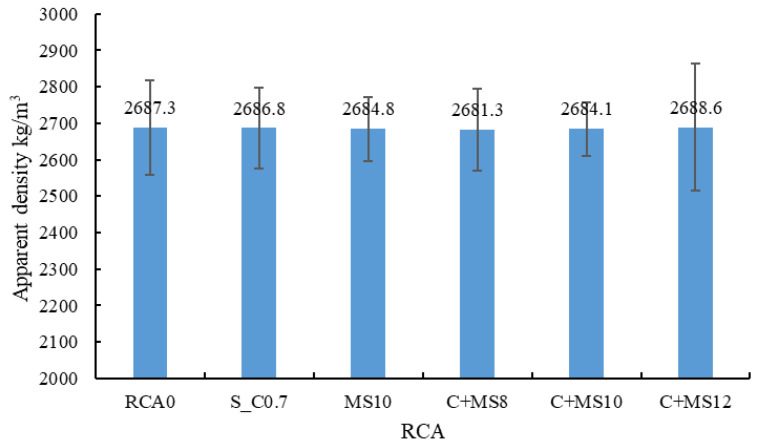
Apparent density of the treated RCA.

The apparent density results are presented in Figure 9. The apparent density of the RCA was not significantly affected by the treatment methods, indicating that neither the cement slurry nor the MSS solution increased the density of the RCA. The possible reasons are as follows: (i) the penetration depth of the cement slurry into the old mortar is limited; (ii) the layer of hardened cement paste formed on the surface of the treated RCA is relatively thin and possesses a low density [40]; and (iii) treatment with the MSS solution generates a molecular film on the surface and within the capillary pores of the RCA [52].

(2) Crushing value

**Figure 10:**
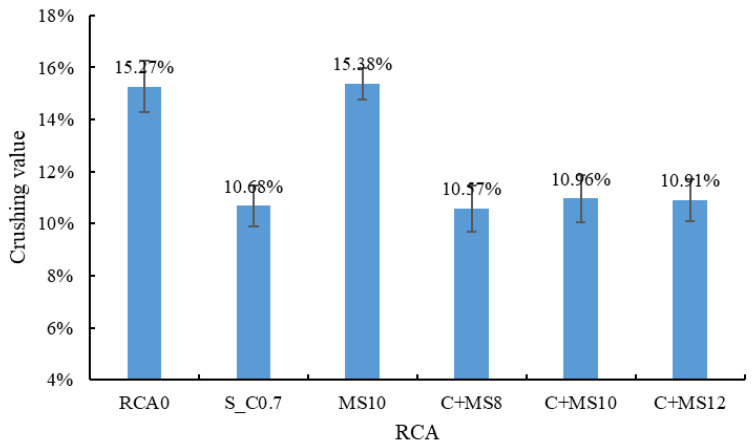
Crushing value of the treated RCA.

The results of the crushing value test are shown in Figure 10. The strength of the RCA was significantly improved by the cement slurry treatment, with the crushing value decreasing by 30.1%. This improvement is attributed to the fact that the hardened cement paste possesses a higher strength than the old mortar, and the old mortar near the surface can also be reinforced through the filling effect of the cement slurry [40,53]. In contrast, the strength of the RCA was not notably affected by the MSS treatment, as only a molecular film is formed on the RCA surface, which does not contribute to enhancing its mechanical strength [52].

(3) Water absorption

**Figure 11:**
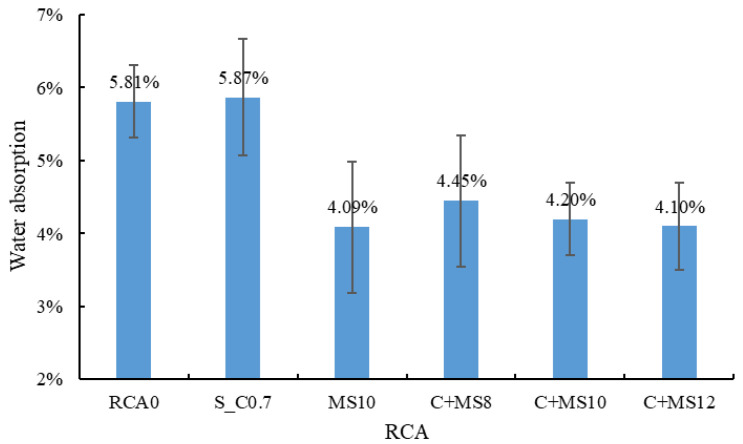
Water absorption of RCA.

Figure 11 presents the water absorption measurement results. Treatment with cement slurry alone did not reduce the water absorption of the RCA, as the hardened cement paste layer formed on the RCA’s surface is also capable of absorbing water [40,53]. In contrast, the use of the MSS solution significantly reduced the water absorption of the treated RCA. Compared to RCA0, the water absorption of MS10, treated with the 10% MSS solution, decreased by 29.6%.

For the combined treatment method, compared to C0.7, the water absorption of C + MS8, C + MS10, and C + MS12 decreased by 24.2%, 28.5%, and 30.1%, respectively. This reduction is primarily attributed to the MSS solution, which reacts with the old mortar to form a hydrophobic layer on the RCA surface, thereby hindering water transport [52]. As the concentration of the MSS solution increased, the water absorption exhibited a decreasing trend. However, when the concentration reached 10%, the rate of decrease became less pronounced, suggesting that an effective and stable hydrophobic layer had formed at this concentration. Therefore, a 10% MSS solution concentration is recommended.

(4) Dynamic water absorption of RCA

**Figure 12:**
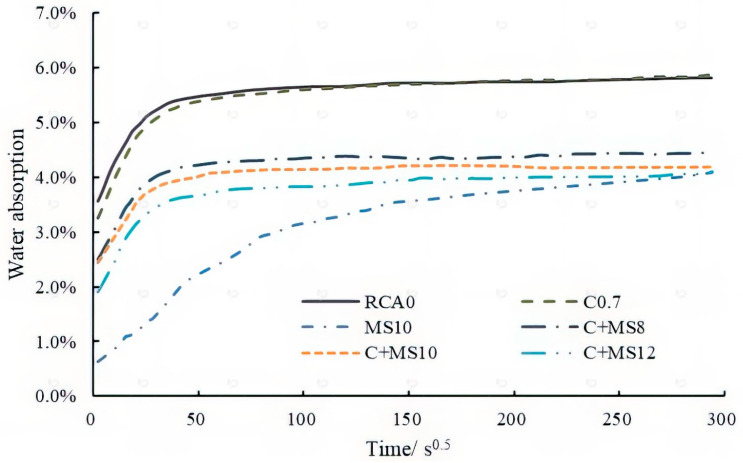
Evolution curves of water absorption of RCAs.

The results of the dynamic water absorption test are shown in Figure 12. All RCAs exhibited a rapid water absorption rate, particularly RCA0 and C0.7. The evolution curves of RCA0 and the RCAs treated with cement slurry—including C0.7, C + MS8, C + MS10, and C + MS12—showed similar patterns. In contrast, when treated with the 10% MSS solution, the water absorption curve of MS10 differed markedly from those of the other RCAs, with a significantly slower water absorption rate.

**Figure 13:**
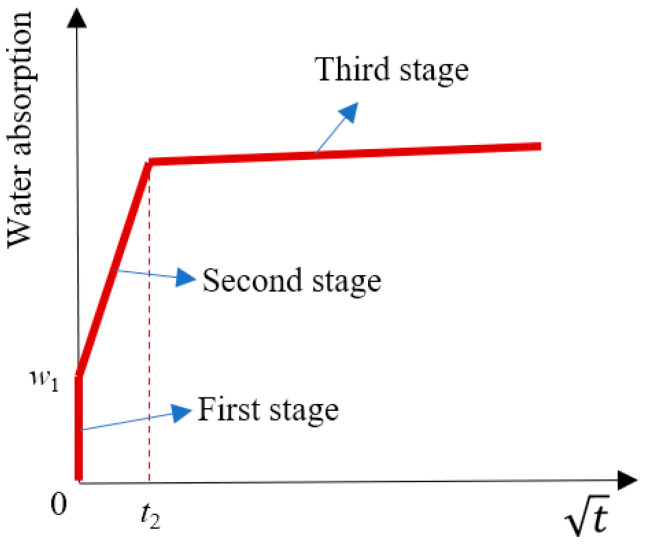
Schematic of the three-stage model [40,41].

Within 24 h, the water absorption behavior of all RCAs displayed a characteristic three-stage pattern. According to [40,41], the schematic diagram of this three-stage model is presented in Figure 13, and the main parameters for each RCA are listed in Table 6. In the first stage, water absorption exhibited an initial leap upon immersion. The saturation degree at the end of this stage (SD1) was approximately 50% for RCA0, C0.7, C + MS8, C + MS10, and C + MS12, whereas SD1 for MS10 was only around 13%. To validate the effectiveness of the three-stage model, the Prediction Interval Check method was employed. The main steps include the following: (1) calculate predicted values using the three-stage model; (2) compute the 95% prediction interval; (3) compare with actual observed values; and (4) calculate the coverage proportion of the 95% prediction interval (CP_95_). The results of CP_95_ were also listed in Table 6. It can be seen that SD1 and SD2 can be well predicted using the three-stage model.

During the second stage, water absorption increased rapidly. By the end of this stage, the saturation degree (SD2) of all RCAs—except MS10—exceeded 90%, indicating that these aggregates were essentially saturated. For MS10, SD2 reached just over 80%. The time required to complete the second stage (*t*_2_) was notably affected by the treatment method. For RCA0 and C0.7, *t*_2_ was approximately 600 s. When the MSS solution was applied, *t*_2_ increased, particularly for MS10, where it extended to about 9000 s, demonstrating the significant inhibitory effect on water transport.

The slope of the second stage reflects the water absorption rate (WAR). The WARs for RCA0 and C0.7 were 0.0009 and 0.001, respectively, indicating that the cement slurry treatment alone did not substantially affect the water absorption rate. In contrast, the WAR for MS10 was reduced to 0.0003, indicating a pronounced inhibition of water transport. For the combined treatments C + MS8, C + MS10, and C + MS12, the WARs were approximately 0.0006, suggesting that the inhibitory effect of the combined treatment was intermediate between the two individual treatments.

Finally, in the third stage, the water absorption rates of all the RCAs gradually stabilized, marking the approach to saturation equilibrium.

(5) SEM and EDS

**Figure 14:**
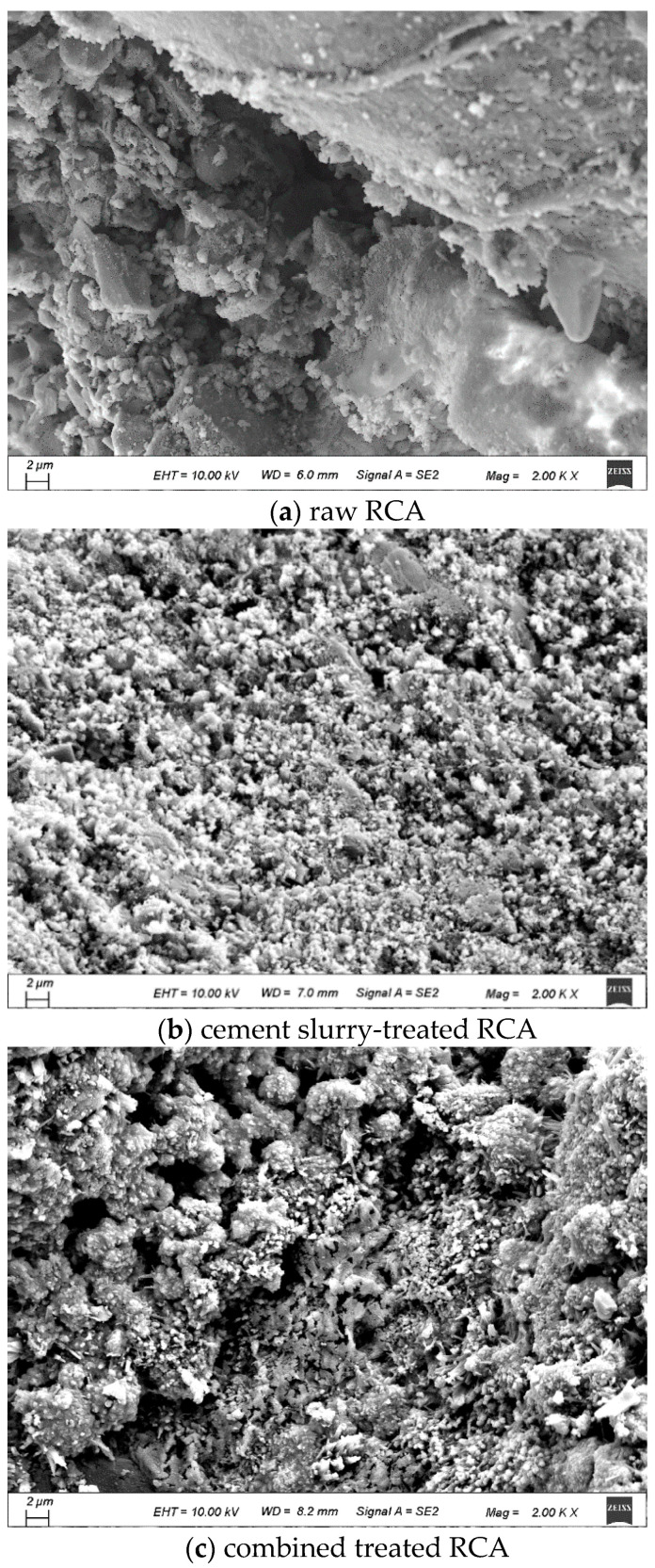
Micro-morphologies of RCA.

The micro-morphologies of the treated RCA and untreated RCA are presented in Figure 14. In comparison, the untreated RCA exhibits a rough and porous surface, whereas after treatment with cement slurry, the surface roughness of the RCA decreases significantly, and the pores become notably smaller, as reported in [40]. This is because the cement slurry adheres to the surface of the RCA, filling the pores and cracks, thereby resulting in a smoother RCA surface. Nevertheless, minute pores remain observable on the treated RCA surface. The aforementioned mechanisms collectively demonstrate that while cement slurry treatment improves the mechanical strength of the RCA, it exhibits constrained efficacy in mitigating water absorption due to persistent microporosity. When treated with the MSS solution, the RCA exhibited no significant morphological changes at the microscale. This indicates that the reduction in water absorption is not achieved through pore blockage, but rather through the formation of a hydrophobic molecular film on the RCA surface, which effectively inhibits water penetration into the RCA.

**Figure 15:**
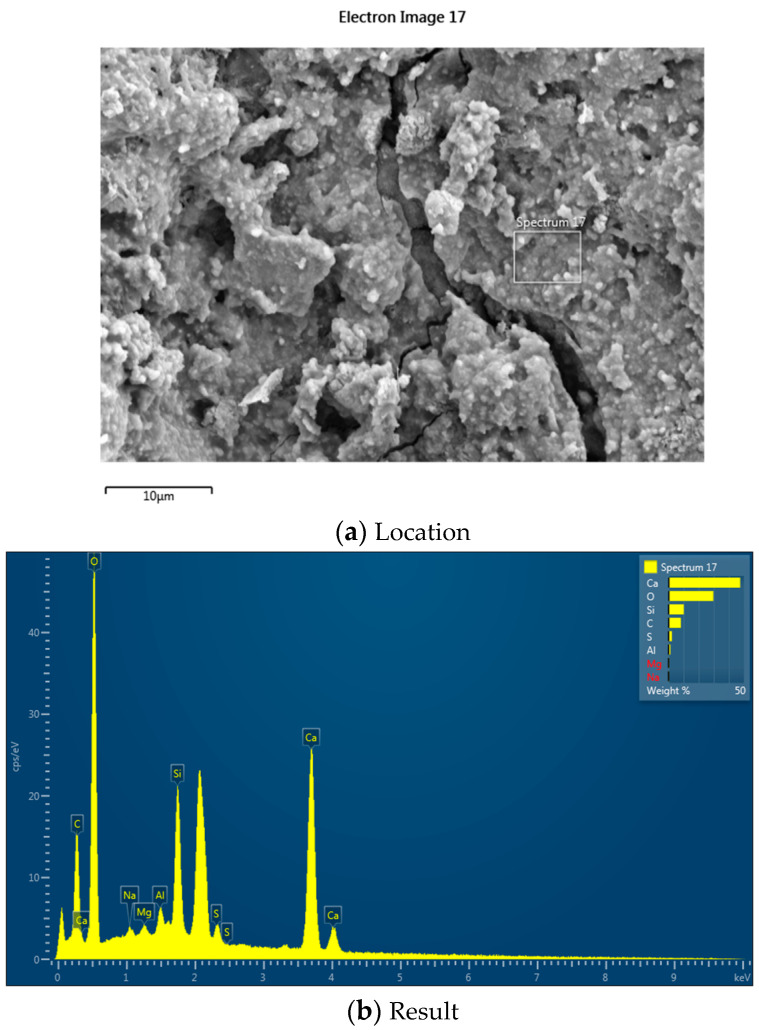
EDS result of the combined treated RCA.

According to the EDS results (Figure 15 and [41]), compared to the untreated RCA, the C element content of the RCA treated with cement slurry and the MSS solution showed a significant decrease. This indicates that the treated RCA surface is coated with a new layer of cement paste, and the newly hardened cement paste layer enhances the RCA’s performance. After composite treatment, the Si element content did not increase significantly, suggesting that the MSS solution penetrated into the RCA without forming a concentrated layer on the surface. This demonstrates that the reduction in the water absorption of the RCA by the MSS is not achieved through pore clogging.

(6) Contact angle of old mortar

**Figure 16:**
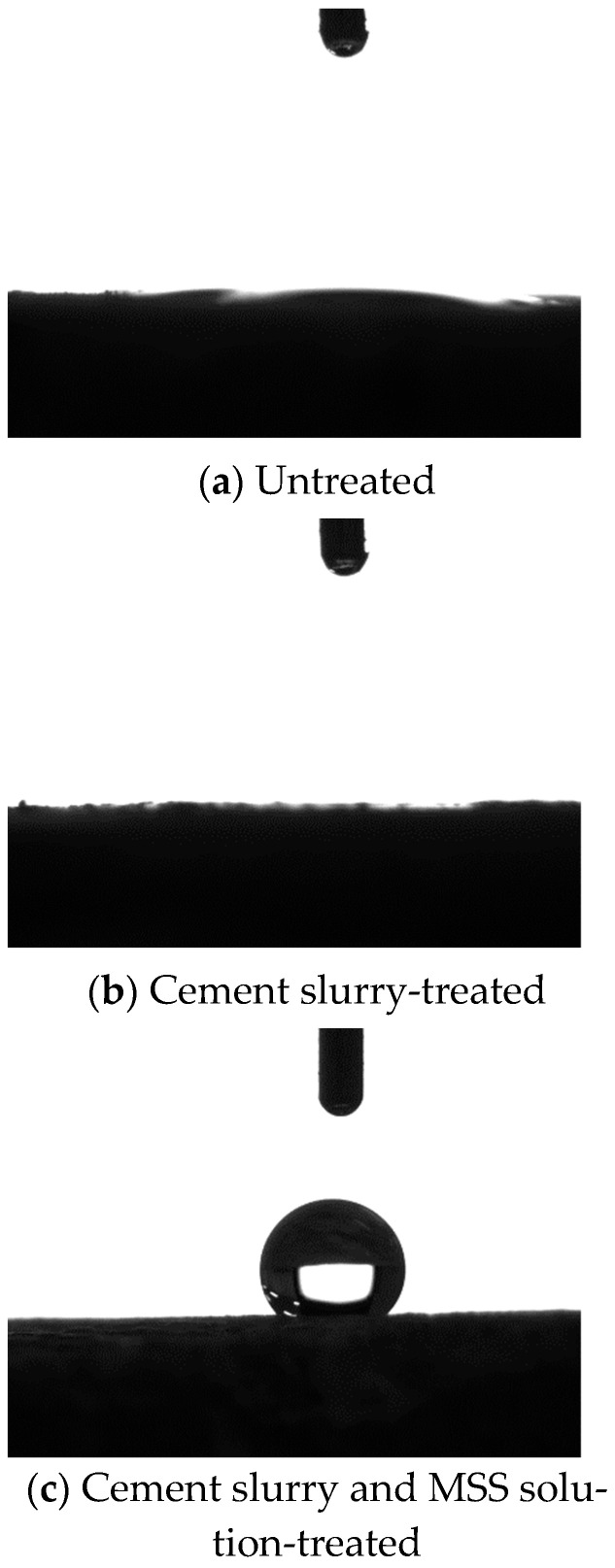
Water drops on the old mortar.

Water drops on the old mortars are shown in Figure 16. Obviously, the untreated mortar and the cement slurry-treated mortar were hydrophilic and the water drops disappeared quickly, as shown in Figure 16a,b. When the MSS solution was used, as shown in Figure 16c, the combined treated mortar was hydrophobic and the contact angle was 136.9°. According to the Young–Laplace function, the capillary pressure was as follows [54]:

(7) $$P_{gl}\;=\;\frac{2\gamma cos\theta }{r}$$

where $P_{gl}$ is the capillary pressure, θ is the contact angle, r is the capillary radius, and γ is the surface tension of water. When θ exceeds 90°, $P_{gl}$ is negative, which is called anti-capillary, meaning the transport of water is inhibited.

(7) FTIR result

**Figure 17:**
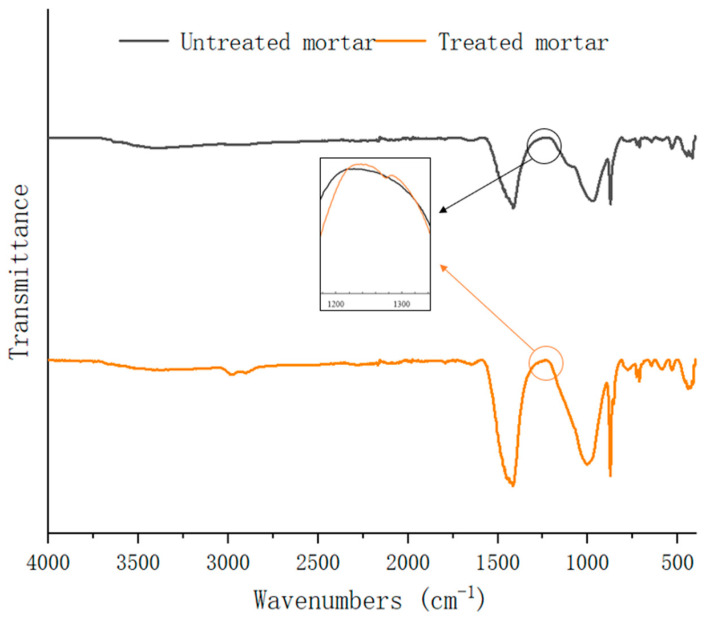
FTIR spectrum.

The infrared spectrum result is shown in Figure 17. The two FTIR spectra were basically the same. The absorption band around 970 cm^−1^ was Si-O-Si. The absorption bands around 1421 cm^−1^ and 870 cm^−1^ were CaCO_3_ which meant that carbonization occurred in the old mortar. For the MSS solution-treated sample, the weak absorption bands around 1270 cm^−1^, 2910 cm^−1^, and 2980 cm^−1^ were observed, indicating the formation of CH_3_ in the sample, which meant that the sample possessed hydrophobic characteristics.

### 3.2. Results of Concrete Tests

(1) Compressive strength

**Figure 18:**
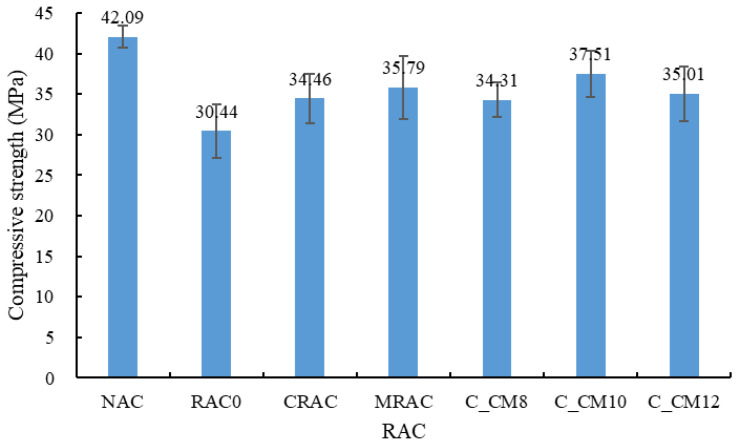
Compressive strength of concrete.

The compressive strength results are shown in Figure 18. The NAC showed the highest compressive strength. When the NCA was completely replaced by the untreated RCA, the compressive strength of RAC0 decreased by 27.7% and reached 30.44 MPa. When the treated RCAs were used, the compressive strength was enhanced. The compressive strength of the CRAC and MRAC increased by 13.2% and 17.6%, respectively, compared to RAC0. As mentioned before, the MSS solution has no obvious influence on the strength of the RCA. Therefore, the enhancement of compressive strength was due to the improvement of the interfacial zone between the RCA and the new mortar. When using the combined treatment method, the compressive strength changed with the concentration of the MSS solution. Namely, 10% was the best concentration here and the compressive strength of C_CM10 increased by 23.2%. According to [55], the MSS could react with cement and lead to the formation of expansive products that could enhance the concrete. Here, the MSS adhered to the surface of the RCA and the adjacent cement mortar was influenced. At a lower concentration, the enhancement effect was not obvious, and the compressive strength of C_CM8 was basically equal to that of the CRAC. At a higher concentration, more expansive products were formed, which had an adverse effect on the compressive strength of the RAC and the compressive strength of C_CM12 was lower than C_CM10.

(2) Splitting tensile strength

**Figure 19:**
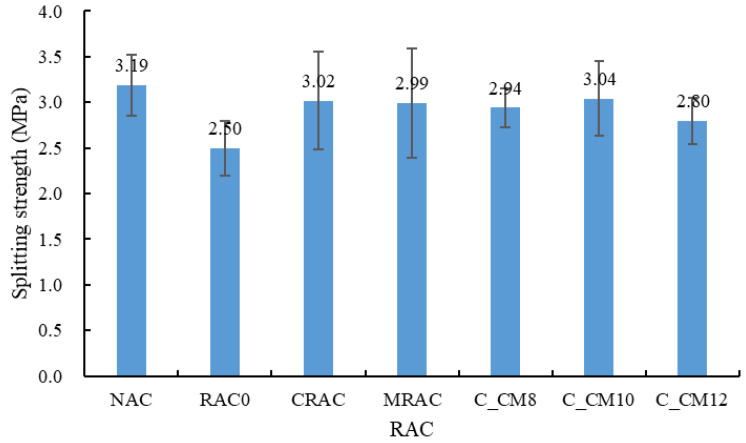
Splitting strength of concrete.

The splitting tensile strength results of the concrete are presented in Figure 19. Similarly to the trend observed for compressive strength, the NAC exhibited the highest splitting tensile strength, while RAC0 showed the lowest. When the NCA was replaced with the untreated RCA, the splitting tensile strength decreased by 21.6%. However, a noticeable improvement was observed when the treated RCAs were used. Specifically, the splitting tensile strength of the CRAC and MRAC increased by 20.7% and 19.4%, respectively, compared to RAC0. Among the combined treated samples, C_CM10 achieved the highest splitting tensile strength, with an increase of 21.6% relative to RAC0.

(3) Water absorption

**Figure 20:**
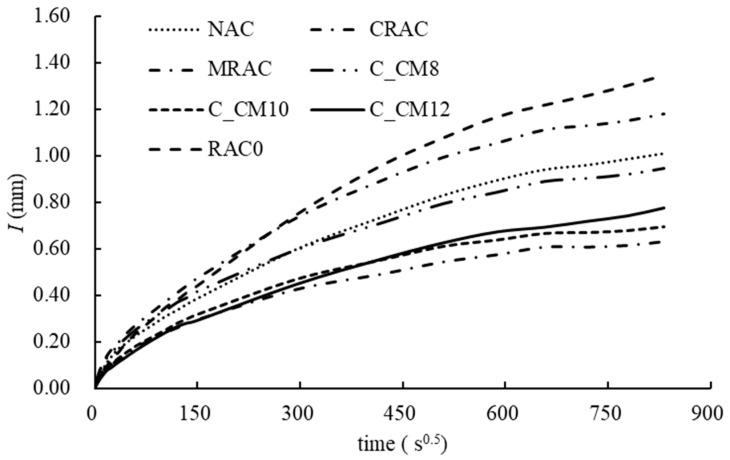
Evolution of water absorption of concrete.

The water absorption results are shown in Figure 20. RAC0 exhibited the highest water absorption, followed by the CRAC (the RCA treated with cement slurry only), with the NAC showing the lowest value. These results indicate that the use of untreated RCA can lead to reduced concrete durability, while the application of cement slurry-treated RCA can partially improve durability, although the water absorption of the treated RCA remains higher than that of the NAC. When MSS solution-treated RCA was used, durability was further enhanced, with water absorption values lower than that of the NAC. Among these, the MRAC achieved the best performance, with water absorption reduced by 53.3% compared to RAC0. For the combined treated samples, C_CM10 also demonstrated a notable improvement, though its absorption was still slightly higher than that of MRAC.

**Figure 21:**
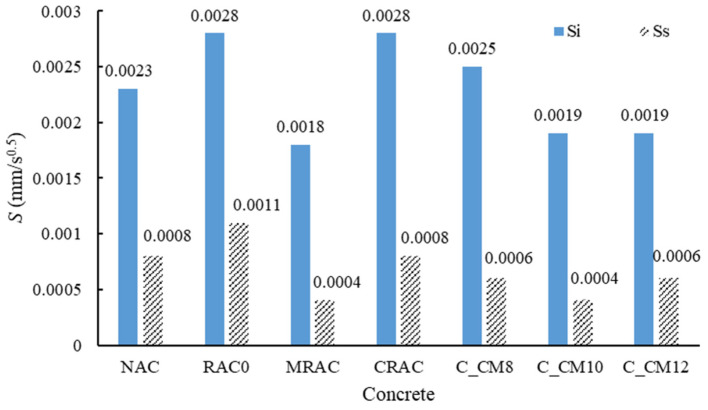
Absorption rate of concrete.

The *S*_i_ and *S*_s_ results for all samples are presented in Figure 21. Both *S*_i_ and *S*_s_ of the NAC were lower than those of RAC0, indicating that replacing the NCA with the untreated RCA increased the water absorption rate. RAC0 and the CRAC exhibited the fastest initial absorption rates, suggesting that the cement slurry treatment alone did not significantly affect the Si value. However, the *S*_s_ value of the CRAC was lower than that of RAC0, indicating a reduction in the second-stage absorption rate.

When MSS-treated RCAs were used—particularly at concentrations exceeding 10%—the water absorption rates decreased substantially. Compared with RAC0, the *S*_i_ and *S*_s_ values of C_CM10 were reduced by 32.1% and 63.6%, respectively, and were also lower than those of the NAC. Previous studies have reported that incorporating the MSS into concrete can effectively reduce its water absorption [56]. In this study, the MSS was introduced through the treated RCA, resulting in an anti-capillary effect predominantly at the interface between the RCA and the new mortar, thereby inhibiting water transport in that region.

(4) Nanoindentation

**Figure 22:**
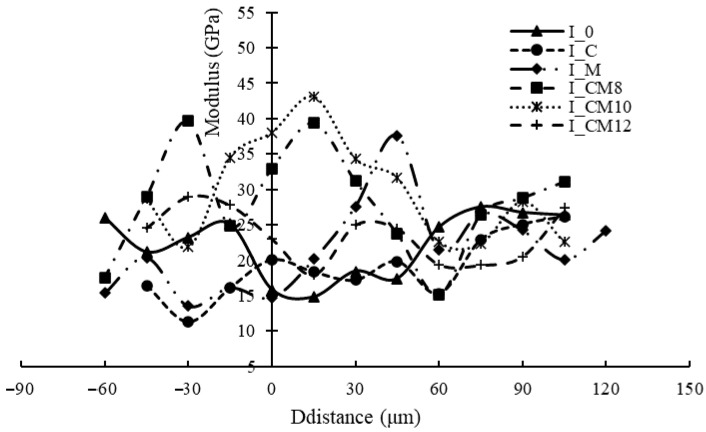
Nanoindentation result of ITZ.

The nanoindentation results are presented in Figure 22. For I_0 and I_C, a lower modulus zone was observed in the new mortar adjacent to the RCA, corresponding to the interfacial transition zone (ITZ), as previously identified in [6]. The width of the ITZ was approximately 45 μm for I_0 and 60 μm for I_C.

When the MSS solution was applied, the mechanical properties of the ITZ changed significantly, with a notable enhancement reflected by a higher modulus compared to the surrounding new mortar. For I_M and I_CM12, the modulus increased with distance from the interface, reaching a peak at around 30–50 μm before subsequently decreasing. In contrast, for I_CM8 and I_CM10, the modulus reached its maximum value closer to the interface, at around 15 μm, before declining.

These results indicate that the combined treatment, particularly with appropriate MSS concentrations, can effectively densify and strengthen the ITZ, improving the mechanical performance in the interfacial region.

**Figure 23:**
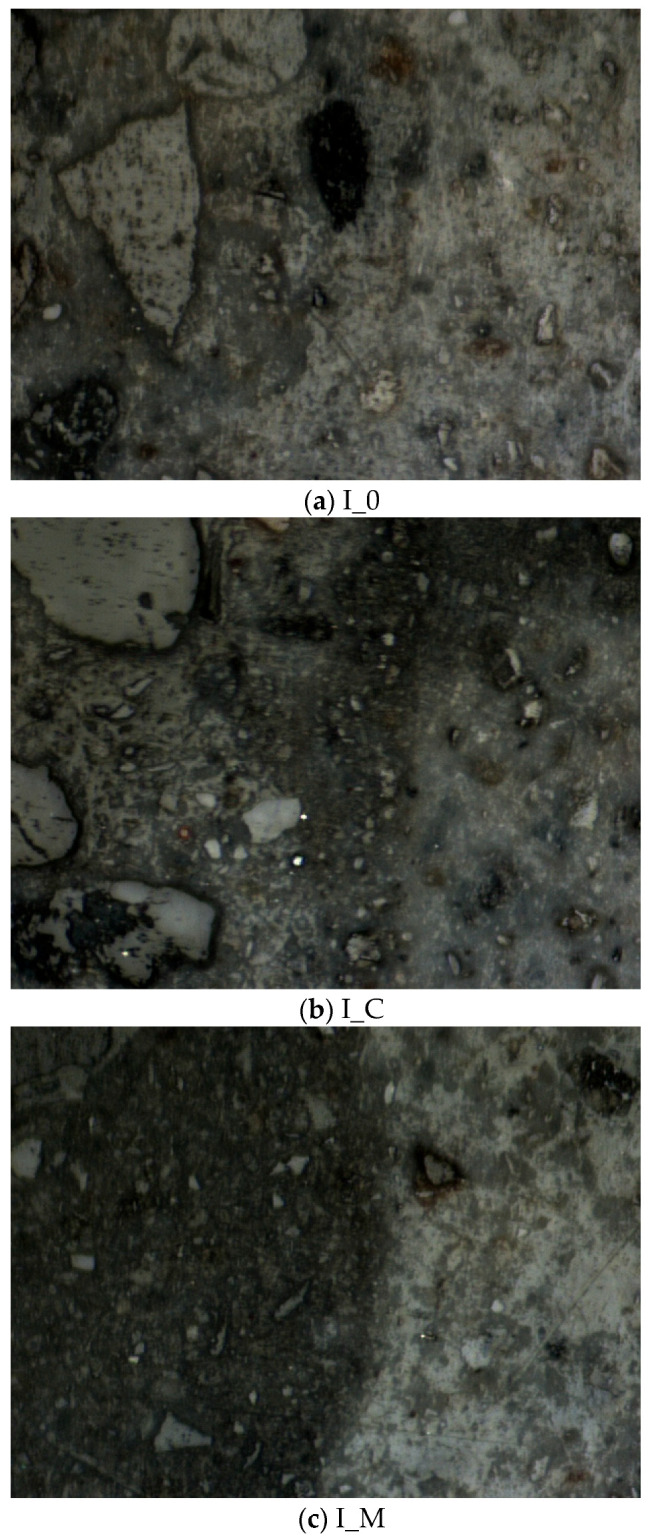

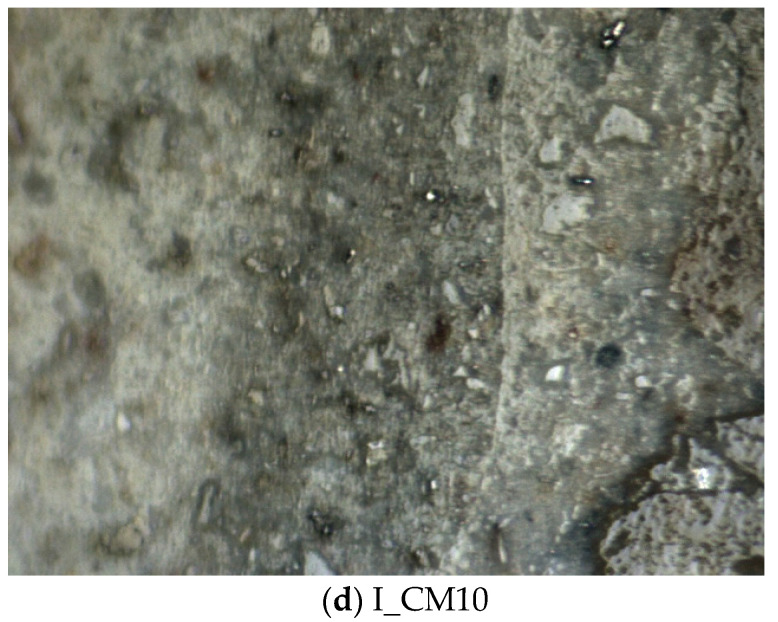
ITZ morphology.

The micro-morphologies of the ITZs recorded by the nanoindentation instrument are shown in Figure 23. In both I_C and I_CM10, an intermediate layer was observed between the RCA and the new mortar, corresponding to the hardened cement slurry formed during the cement slurry treatment of the RCA. While this hardened cement slurry layer made the surface of the treated RCA smoother, it also increased the structural complexity of the RAC, which could potentially have adverse effects on the overall performance of the concrete.

## 4. Discussion

As revealed in the study, RCAs inherently contain adhered cement mortar, exhibiting a porous, rough surface morphology with abundant microcracks (Figure 14a). These characteristics directly contribute to their high water absorption and elevated crushing values, resulting in inferior overall performance [57,58]. Consequently, such subpar RCA quality adversely impacts the mechanical and durability properties of the RAC [59].

**Figure 24:**
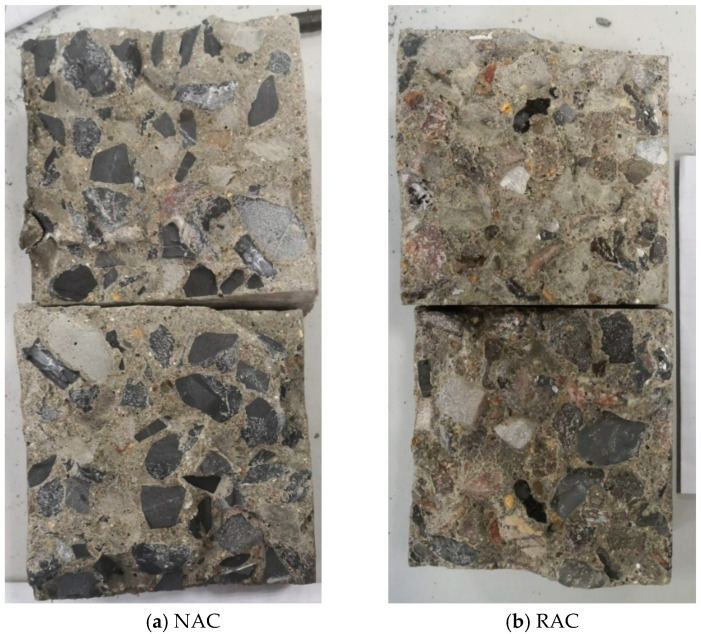
Failure image of concrete in the splitting tensile test.

The images of specimen failure in the splitting tensile test are shown in Figure 24. For natural aggregate concrete (NAC), more cases of aggregate fracture were observed, indicating a relatively strong interfacial bond between the natural aggregates and the mortar matrix. In contrast, for recycled aggregate concrete (RAC), more instances of detachment between the recycled aggregates and the mortar matrix were observed, representing interfacial failure. As reported in [60,61], in the RAC, the interfacial transition zone between the recycled aggregate and the mortar matrix is the weakest link. This suggests that enhancing the interfacial performance between recycled aggregates and the mortar matrix is more beneficial for improving the mechanical properties of the RAC. Similar phenomena have been consistently reported in [62,63], corroborating our findings.

When cement slurry was used to treat the RCA, a hardened layer forms on the RCA surface (Figure 2), filling pores and cracks, thereby increasing the strength of the treated RCA [53] and making its surface smoother [64]. However, capillary pores remained visible, and the hardened layer was hydrophilic (Figure 14b). As a result, the water absorption of the treated RCA did not decrease [53]. When the cement slurry-treated RCA was incorporated into the RAC, the mechanical property of the ITZ between the RCA and the new mortar was slightly enhanced, resulting in an increase in compressive strength and splitting tensile strength of the RAC. However, the hardened cement coating increases the complexity of the interfacial transition zone (ITZ) in the RAC (Figure 23d). According to [60,65], the weak ITZ is a critical factor limiting the performance of recycled aggregate concrete (RAC). Consequently, when cement slurry-treated RCA is used in the RAC, its performance remains constrained by the resulting complex ITZ microstructure.

In contrast, when the MSS solution was employed to treat the RCA, there was no significant effect on the strength of the RCA, but the water absorption rate of the RCA showed a noticeable decrease. When cement slurry and the MSS solution were applied in combination, the treated RCA exhibited improved comprehensive properties, including both increased strength and reduced water absorption. This is because the MSS is a novel waterproofing material that forms a hydrophobic molecular film on the surface of cement-based materials [46,66,67].

When MSS-treated RCA is incorporated into the RAC, both the mechanical properties and capillary water resistance of the RAC are enhanced. This improvement occurs because of the following: (1) The MSS forms hydrophobic films within RCA, effectively inhibiting water transport through RAC. (2) The MSS coating on RCA surfaces chemically reacts with the new mortar to produce expansive products, thereby reinforcing the interfacial transition zone between the RCA and the new mortar, which consequently enhances the RAC’s mechanical performance [55]. Notably, when the MSS solution concentration exceeds 10%, RAC strength decreases due to excessive expansive products generating microcracks in the interfacial transition zone [55,56].

## 5. Conclusions

Based on the concept of composite modification, this study proposes a method for modifying recycled concrete aggregate (RCA) using a combination of cement slurry and an MSS solution. From the perspectives of micromorphology, functional groups, and surface energy, the mechanism by which the composite modification method influences the properties of the RCA was elucidated. Furthermore, from the viewpoint of the interfacial transition zone, the mechanism through which the composite-modified RCA affects the performance of recycled aggregate concrete (RAC) was revealed. Based on the abovementioned results, the following conclusions can be drawn:

- The combined spraying method using both cement slurry and the MSS solution not only enhanced the strength of the RCA but also reduced its water absorption, thereby improving the overall performance of the treated RCA. The strength enhancement of the RCA primarily relies on the encapsulating and pore-filling effects of the cement slurry, while the reduction in water content is mainly attributed to the formation of a hydrophobic membrane on the RCA surface by the MSS solution.
- The compressive strength and splitting tensile strength of the RAC were improved by incorporating either cement slurry-treated RCA or MSS-treated RCA. In particular, the water absorption of the RAC was significantly reduced when MSS-treated RCA was used.
- Compared with RAC prepared with MSS-treated RCA alone, the RAC incorporating combined treated RCA exhibited further improvements in compressive strength and splitting tensile strength. However, no further reduction in water absorption was observed. Overall, a 10% MSS solution concentration is recommended based on performance optimization.
- The MSS treatment effectively enhanced the mechanical properties of the interfacial transition zone (ITZ) between the treated RCA and the new mortar—widely regarded as the weakest region in RAC—and this enhancement was influenced by the concentration of the MSS solution. Additionally, it is worth noting that when cement slurry-treated RCA was used, an interlayer was observed near the ITZ, which could increase the structural complexity of RAC.
- The composite modification method not only enhances the mechanical properties of the RAC but also demonstrates superior performance in reducing capillary water absorption. Therefore, the research findings can be applied to engineering projects affected by capillary water, such as underground structures or marine environments subject to wave action.
- Future research should focus on the reactions between the MSS, old mortar, and new mortar, further clarifying the influence of the MSS on the RCA and the interface between old and new mortar from the perspective of reaction products. Additionally, the practical application of these findings in real-world engineering projects is necessary to validate their effectiveness under actual working conditions.

## Figures and Tables

**Table 1:** Chemical composition of the cement(wt.%).

Composition	CaO	SiO_2_	Al_2_O_3_	Fe_2_O_3_	MgO	SO_3_
Percentage	59.26	20.47	6.31	4.08	2.01	2.23

**Table 2:** The treatment scheme of RCA.

Name	*w*/*c* of Cement Slurry	Concentration of MSS Solution
RCA0	-	-
C0.7	0.7	-
MS10	-	10%
C + MS8	0.7	8%
C + MS10	0.7	10%
C + MS12	0.7	12%

**Table 3:** Mixture proportion of concrete (per m^3^).

Coarse Aggregate/kg	Cement/kg	Sand/kg	Water/kg
1003	365	821	182.5

**Table 4:** The research scheme of concrete.

Name	Coarse Aggregate	Ratio of RCA Replacement
NAC	NA	0
RAC0	RCA0	100%
CRAC	C0.7	100%
MRAC	MS10	100%
C_CM8	C + MS8	100%
C_CM10	C + MS10	100%
C_CM12	C + MS12	100%

**Table 5:** Research scheme of nanoindentation.

Name	I_0	I_C	I_M	I_CM8	I_CM10	I_CM12
RCA type	RCA0	C0.7	MS10	C + MS8	C + MS10	C + MS12

**Table 6:** Main parameters of the three-stage model of RCAs.

RCA	Function of the Second Stage	*w*_1_ (%)	Function of the Third Stage	*t*_2_ (s)	*SD*_1_ (%)	*SD*_2_ (%)
RCA0	*w*_at_ = 0.0009$\sqrt{t}$ + 0.0336 R^2^ = 0.995	3.36	*w*_at_ = 9 × 10^−6^$\sqrt{t}$ + 0.0557 R^2^ = 0.9598	615	57.8% (100%)	96.3% (83.3%)
C0.7	*w*_at_ = 0.001$\sqrt{t}$ + 0.0301 R^2^ = 0.996	3.01	*w*_at_ = 1 × 10^−5^$\sqrt{t}$ + 0.0549 R^2^ = 0.9831	628	51.3% (100%)	94.0% (100%)
MS10	*w*_at_ = 0.0003$\sqrt{t}$ + 0.0053 R^2^ = 0.9886	0.53	*w*_at_ = 4× 10^−5^$\sqrt{t}$ + 0.0301 R^2^ = 0.9959	9098	13.0% (100%)	82.9% (100%)
C + MS8	*w*_at_ = 0.0006$\sqrt{t}$ + 0.0254 R^2^ = 0.9939	2.54	*w*_at_ = 9 × 10^−6^$\sqrt{t}$ + 0.042 R^2^ = 0.872	789	57.1% (100%)	95.0% (83.3%)
C + MS10	*w*_at_ = 0.0006$\sqrt{t}$ + 0.0232 R^2^ = 0.9929	2.32	*w*_at_ = 2 × 10^−6^$\sqrt{t}$ + 0.0415 R^2^ = 0.8406	936	55.2% (100%)	99.0% (100%)
C + MS12	*w*_at_ = 0.0006$\sqrt{t}$ + 0.0197 R^2^ = 0.9876	1.97	*w*_at_ = 7 × 10^−6^$\sqrt{t}$ + 0.0385 R^2^ = 0.8769	1005	48.0% (100%)	94.4% (83.3%)

Note: The values in parentheses represent the coverage proportion of the 95% prediction interval.

## Data Availability

The original contributions presented in this study are included in the article. Further inquiries can be directed at the corresponding author.
